# The Shear Stress-Induced Transcription Factor KLF2 Affects Dynamics and Angiopoietin-2 Content of Weibel-Palade Bodies

**DOI:** 10.1371/journal.pone.0038399

**Published:** 2012-06-08

**Authors:** Ellen L. van Agtmaal, Ruben Bierings, Bieuwke S. Dragt, Thomas A. Leyen, Mar Fernandez-Borja, Anton J. G. Horrevoets, Jan Voorberg

**Affiliations:** 1 Department of Plasma Proteins, Sanquin-AMC Landsteiner Laboratory, University of Amsterdam, Amsterdam, The Netherlands; 2 Department of Physical Biochemistry, National Institute for Medical Research, London, United Kingdom; 3 Department of Molecular Cell Biology and Immunology, VU Medical Center, Amsterdam, The Netherlands; 4 Department of Molecular Cell Biology, Sanquin-AMC Landsteiner Laboratory, Amsterdam, The Netherlands; Leiden University Medical Center, The Netherlands

## Abstract

**Background:**

The shear-stress induced transcription factor KLF2 has been shown to induce an atheroprotective phenotype in endothelial cells (EC) that are exposed to prolonged laminar shear. In this study we characterized the effect of the shear stress-induced transcription factor KLF2 on regulation and composition of Weibel-Palade bodies (WPBs) using peripheral blood derived ECs.

**Methodology and Principal Findings:**

Lentiviral expression of KLF2 resulted in a 4.5 fold increase in the number of WPBs per cell when compared to mock-transduced endothelial cells. Unexpectedly, the average length of WPBs was significantly reduced: in mock-transduced endothelial cells WPBs had an average length of 1.7 µm versus 1.3 µm in KLF2 expressing cells. Expression of KLF2 abolished the perinuclear clustering of WPBs observed following stimulation with cAMP-raising agonists such as epinephrine. Immunocytochemistry revealed that WPBs of KLF2 expressing ECs were positive for IL-6 and IL-8 (after their upregulation with IL-1β) but lacked angiopoietin-2 (Ang2), a regular component of WPBs. Stimulus-induced secretion of Ang2 in KLF2 expressing ECs was greatly reduced and IL-8 secretion was significantly lower.

**Conclusions and Significance:**

These data suggest that KLF2 expression leads to a change in size and composition of the regulated secretory compartment of endothelial cells and alters its response to physiological stimuli.

## Introduction

Endothelial cells (ECs) are subjected to blood-flow generated laminar shear stress. The laminar flow in blood vessels is pulsatile and can reach shear stress levels of 10 to 70 dyne/cm^2^ [1], [2]. High shear stress induces an atheroprotective endothelial phenotype while absence of shear stress, as occurs near bends and at bifurcations, leads to endothelial dysfunction, characterized by a reduction in barrier function and upregulation of pro-inflammatory gene expression [2], [3], [4]. These sites of disturbed blood flow are more prone to atherosclerotic lesion development [2], [5]. It is well-established that hemodynamic forces have a considerable impact on vascular ECs [2]. One of the transcription factors that are induced by hemodynamic forces is Krüppel-like factor 2 (LKLF, KLF2), which was found to be absent from atheroprone vascular regions and may be considered atheroprotective [6]. Increased expression of KLF2 is also induced by 3-hydroxy-3-methyl-glutaryl-CoA reductase inhibitors (statins) while inflammatory cytokines are found to reduce transcription of KLF2 [7], [8]. Ectopic expression of KLF2 induces both functional and morphological changes in endothelial cells which mimic the effects of shear stress. KLF2 was shown to affect the expression of vascular tone regulating genes which enables the establishment of a functionally quiescent endothelium [3]. ECs expressing KLF2 display anti-inflammatory, anti-thrombotic, anti-migratory, anti-fibrotic and anti-oxidant properties [9], [10].

A number of thrombotic and inflammatory mediators originate from EC-specific, elongated secretory organelles called Weibel-Palade bodies (WPBs). WPBs function as storage vesicle for von Willebrand Factor (VWF), a multimeric glycoprotein which plays a crucial role in platelet plug formation [11], [12], [13]. In addition, these organelles also contain other bioactive compounds including P-selectin [14], [15], lamp3 [16], Ang2 [17], IL-8 [18], [19], eotaxin-3 [20], osteoprotegerin-1 [21] and endothelin-1 [22], the release of which enables the endothelium to actively participate in inflammatory responses, angiogenesis and regulation of vascular tone. Upon stimulation of the ECs with agonists that raise Ca^2+^ or cAMP levels, for example thrombin and epinephrine respectively, WPBs fuse with the plasma membrane resulting in release of their contents in the circulation and exposure of P-selectin on the plasma membrane. However, a subset of WPBs is able to escape regulated exocytosis in response to cAMP-raising agonists and form a perinuclear cluster at the microtubule organizing center (MTOC) [23]. The minus-end directed transport of WPBs along the microtubules to the MTOC is mediated by the dynein/dynactin complex and protein kinase A (PKA) [24].

We have recently shown that expression of KLF2 modulates the thrombin-induced release of WPBs whereas the epinephrine-induced release of these organelles was not affected [3]. In this work we have further characterized the atheroprotective phenotype induced by KLF2 with regard to the secretory pathway of ECs. We show that lentiviral expression of KLF2 leads to an altered morphology and composition of WPBs and results in impaired regulated secretion of Ang2 and (to a lesser extent) IL-8. Strikingly, we found that KLF2 expressing cells no longer display perinuclear clustering of WPBs after stimulation with cAMP-mediated agonists. Our data indicate that the atheroprotective phenotype of KLF2 expressing ECs extends to their regulated secretory pathway and markedly alters the composition and regulation of their secretory response.

## Materials and Methods

### Reagents and Antibodies

EGM-20 medium, epinephrine, thrombin, phorbol 12-myristate 13-acetate (PMA), forskolin and 3-isobutyl-1-methylxanthine (IBMX) were from Sigma Aldrich (Steinheim, Germany). Mouse monoclonal anti-α-tubulin antibody and mouse IgG1 anti γ-tubulin were also from Sigma. Rabbit anti-β-catenin (sc-7199) antibody was from Santa Cruz Biotechnology (Santa Cruz, CA). Mouse monoclonal anti-human angiopoietin-2, mouse monoclonal anti-IL-6 and mouse monoclonal anti-OPG antibodies were from R&D (Minneapolis, MN). Rabbit polyclonal anti-P-selectin antibody was obtained from BD Biosciences, (San Jose, USA). The rabbit polyclonal anti-KLF2 antibody has been described previously [3]. The sheep anti-mouse HRP antibody and the donkey anti-rabbit HRP antibody were from GE Healthcare (Hoevelaken, the Netherlands). Alexa 405-, 488-, 568-, 633-conjugated secondary antibodies were from Molecular Probes (Breda, the Netherlands). IL-1β was from Strathman Biotech (Hannover, Germany). Chemiluminescence blotting substrates were from Roche Diagnostics (Mannheim, Germany). The duoset Ang2 ELISA kit was from R&D. PeliKine human IL-8 and IL-6 ELISA kits were purchased from Sanquin (Amsterdam, the Netherlands). Mouse monoclonal anti-IL-8 was frome Sanquin (Amsterdam, the Netherlands). The serum-free (SF) medium contained RPMI and M199 (in a 1∶1 ratio) supplemented with 0.3 g/L L-glutamine (Sigma) and 1% human serum albumin (Sanquin, Amsterdam, the Netherlands) and penicillin and streptomycin (BD Biosciences). All chemicals used were of analytical grade.

### Cell Culture and Agonist Stimulation

Blood outgrowth endothelial cells (BOECs) were isolated as previously described [25] from 50 ml of venous blood that was drawn from healthy anonymous volunteers, with written permission, in accordance with Dutch regulations and approval from Sanquin Ethical Advisory Board. Mock and KLF2 lentivirus were prepared as described previously [26]. Passage 6 BOECs were transduced by a single exposure to KLF2 or mock lentivirus in the presence of 8 µg/ml polybrene and centrifuged for 90 minutes at 1200 rpm and 37°C. After a total incubation time of 4 hours at 37°C, the medium was refreshed. Transduced cells were cultured using the standard cell culture protocols for BOECs. For the stimulation with epinephrine and forskolin, BOECs were grown in 12-wells plates to confluency. Before stimulation, cells were washed two times with serum-free (SF) medium and pre-incubated in SF medium for 1 hour at 37°C. To measure IL-6 and IL-8 release, cells were stimulated with 10 ng/ml IL-1β in EGM-20 medium for 24 hours [18] and pre-incubated with serum-free medium without IL-1β 6 hours prior to stimulation [27]. Stimulation was performed by replacing the pre-incubation medium with SF medium containing 10 µM epinephrine and 100 µM IBMX, 10 µM forskolin and 100 µM IBMX or 50 ng/ml PMA and further incubation for 1 hour at 37°C.

### Western Blotting and Immunofluorescence

Western blot was performed essentially as described previously [28]. Multimeric composition of VWF was analysed as previously decribed [29]. For immunofluorescence KLF2- and mock-transfected BOECs were grown on collagen-coated glass coverslips, fixed with 3.7% para-formaldehyde (PFA) for 10 min at RT and permeabilized with ice-cold methanol for 10 min. WPBs were visualized using the anti-VWF mouse monoclonal antibody CLB-RAg20 (subclass IgG2b) and Alexa 568-conjugated goat anti-mouse IgG2b. The cellular membrane was stained with rabbit polyclonal anti-β-catenin antibody and Alexa 633-conjugated goat anti-rabbit antibody. The MTOC was visualized using the mouse IgG1 monoclonal antibody anti-γ-tubulin and Alexa 488-conjugated goat anti-mouse IgG1. KLF2 was visualized using the rabbit polyclonal antibody anti-KLF2 and Alexa 488-conjugated goat anti-rabbit IgG. Mouse IgG2a CLB-HEC75 directed against CD31 and Alexa 633-conjugated goat anti-mouse IgG2a were used in combination with the KLF2 antibody. Stainings were performed in PBS supplemented with 1% human albumin and 0.02% saponin. Cells were embedded in Vectashield and analyzed by confocal microscopy using a Zeiss LSM510 (Carl Zeiss, Sliedrecht, the Netherlands) equipped with the appropriate filters. Three-dimensional images were generated by making optical sections of 0.25 µm along the Z-axis of the cells (Z-stacks).

### Quantification of WPB Clustering

Image restoration and 3D analysis of z-stacks of single cells was performed using Image Pro Plus 6.1 (Media Cybernetics, Breda, the Netherlands). Staining for CD31 was used to select an area of interest encompassing a single cell. The location of the MTOC was determined by staining for γ-tubulin. The 3D Open filter was applied to separate narrowly connected WPBs. 3D data were rendered to a 3D Gaussian surface using the 3D constructor module to automatically recognize vesicles and the MTOC and to calculate their position. The distance of the individual WPBs to the MTOC was calculated using the following formula: d  =  √((x_WPB_-x_MTOC_)^2^+ (y_WPB_ – y_MTOC_)^2^+ (z_WPB_ – z_MTOC_)^2^), where x, y and z are the spacial coordinates in the 3D model (respectively the width, height and depth).

### Statistics

Differences in WPB length, amount and number of clustered WPBs were analyzed using a Student’s t-test or two-way ANOVA with significance assumed at p<0.05.

## Results

### Expression of KLF2 Results in More but Shorter WPBs

**Figure 1:**
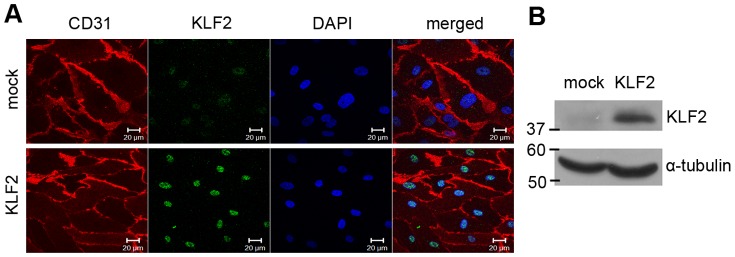
Expression of KLF2 in BOECs. (A) Immunofluorescent analysis of mock- and KLF2-transduced BOECs. Cells were immunostained for CD31 (red) and KLF2 (green); nuclei were stained using DAPI (blue). Scale bars: 20 µm; (B) Western blot analysis of KLF2 expression in lysates of KLF2-transduced and mock-transduced BOECs; α-tubulin is shown as a loading control.

Lentiviral transduction was used to introduce KLF2 into BOECs. Early passage BOECs were transduced with lentivirus containing KLF2 cDNA or empty virus (mock). Cells were cultured for 7 days to allow for the establishment of a steady-state gene expression pattern, as KLF2 effects are induced only after prolonged overexpression (more than 24 hours) [6]. An increased expression of KLF2 in KLF2-transduced cells compared to mock-transduced cells was demonstrated by immunocytochemistry (Figure 1A) and by Western blot analysis (Figure 1B).

**Figure 2:**
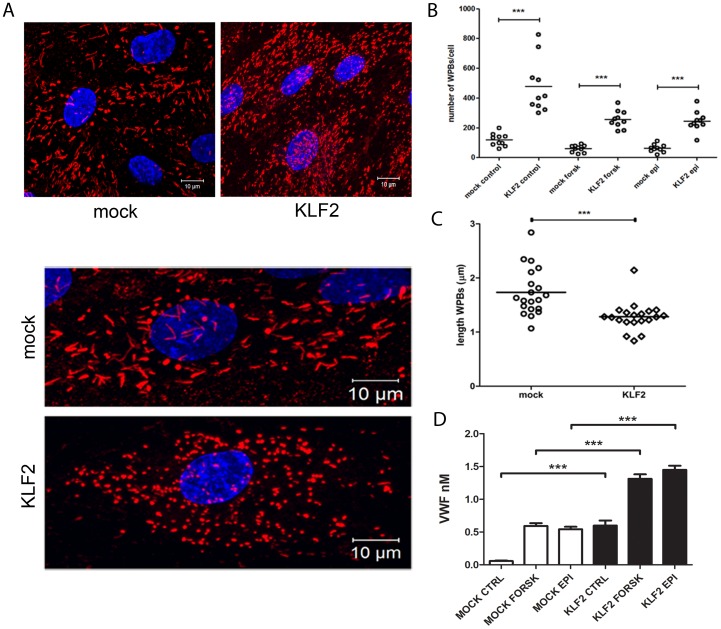
Reduced average length of WPBs in KLF2-transduced BOECs. (A) Confocal microscopy analysis of WPBs in mock- and KLF2-transduced BOECs stained for VWF (red) and DAPI (blue). Scale bars: 10 µm. (B) Average amount of WPBs per cell in unstimulated and stimulated mock-transduced BOECs and KLF2-transduced BOECs. ***P<0.0001 using Student’s t- test (C) The average length of the WPBs in individual mock- or KLF2-transduced BOECs. WPBs from 20 randomly selected cells were analyzed. ***P<0.0005 by Student’s t-test (D) Release of VWF from forskolin/IBMX- and epinephrine/IBMX-stimulated KLF2 (black bars)- and mock (white bars)-transduced BOECs. ***P<0.0001 by Students t-test. Error bars represent SEM.

It has been reported previously that expression of KLF2 increases the secretion of VWF and the average amount of WPBs/cell in HUVECs [3]. Expression of KLF2 in BOECs also resulted in a 4-fold increase in the number of WPBs per cell (Figure 2B). Following stimulation with cAMP raising agonists the average number of WPBs per cell declined 2-fold in both KLF2- and mock-transduced endothelial cells (Figure 2B). Close inspection of the morphology of WPBs in KLF2-transduced cells revealed a slightly altered morphology. The average length of WPBs in KLF2 lentiviral transduced BOECs was 1.3±0.3 µm whereas WPB in mock-transduced BOECs had an average length of 1.7 µm (Figures 2A and C). Less elongated WPBs were frequently observed in KLF2-expressing cells, however most WPBs, although shorter, remained rod-shaped. The forskolin- and epinephrine-stimulated secretion of VWF in the medium of mock- and KLF2-transduced BOECs was determined (Figure 2D). A 2-fold increase in stimulated VWF release in KLF2-transduced cells was observed. Also, an increase in VWF release in the absence of secretagogues was observed in KLF2 expressing cells although the extent of this increased release varied between 0.1 to 0.6 nM among different experiments.

### WPBs Lack Angiopoietin-2 after Expression of KLF2

**Figure 3:**
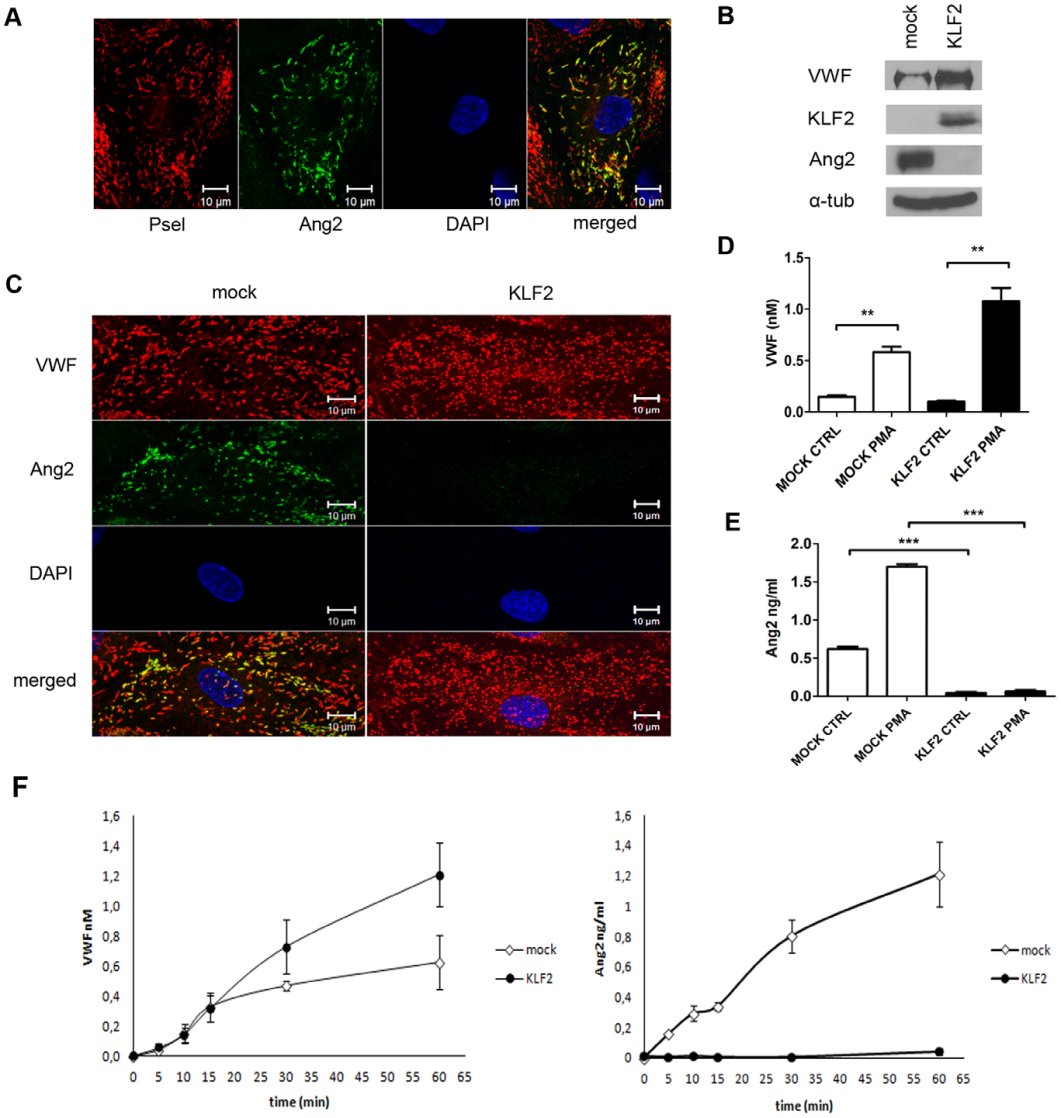
Angiopoietin-2 content of WPBs in mock- and KLF2-transduced BOECs. (A) Immunofluorescence image showing the co-localization of Ang2 (green) and P-selectin (red) in WPBs of mock-transduced BOECs. Nuclei were visualized with DAPI (blue). Scale bars: 10 µm. (B) Western blot analysis of VWF, KLF2 and Ang2 expression in lysates of mock- and KLF2-transduced BOECs; α-tubulin is shown as a loading control. (C) Mock- and KLF2-transduced BOECs stained for VWF (red) and Ang2 (green). Nuclei were stained using DAPI (blue). Scale bars: 10 µm. (D-E) Release of Ang2 and VWF from PMA-stimulated KLF2 (black bars)- and mock (white bars)-transduced BOECs measured by determining the concentration of Ang2 in the medium by ELISA. **P<0.001; ***P<0.0001 by Students t-test. (F) Time course of regulated VWF and Ang2 secretion after PMA stimulation of mock- and KLF2-transduced BOECs.

**Figure 4:**
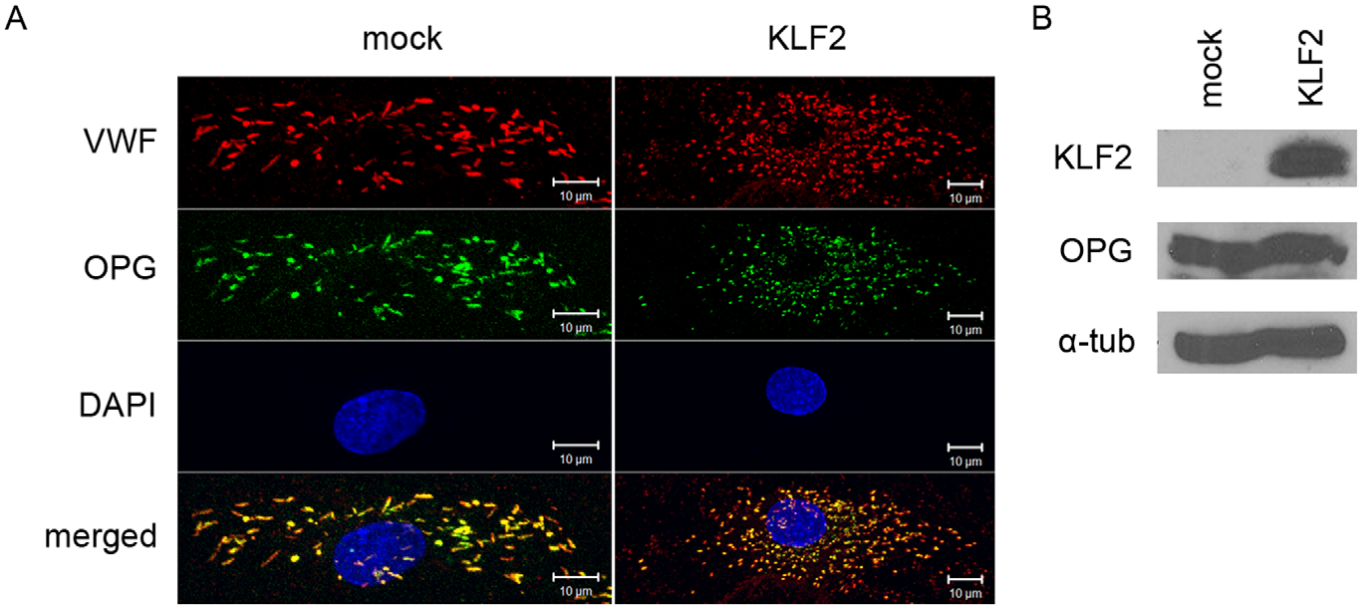
OPG content of mock- or KLF2-transduced BOECs. (A) Immunofluorescence image showing co-localization of OPG (green) and VWF (red) in both mock- and KLF2-tranduced BOECs. Nuclei were stained using DAPI (blue). Scale bars: 10 µm.(B) Western blot analysis for VWF, KLF2, IL-8 and IL-6 expression in lysates of mock- and KLF2-transduced BOECs; α-tubulin was shown as a loading control.

**Figure 5:**
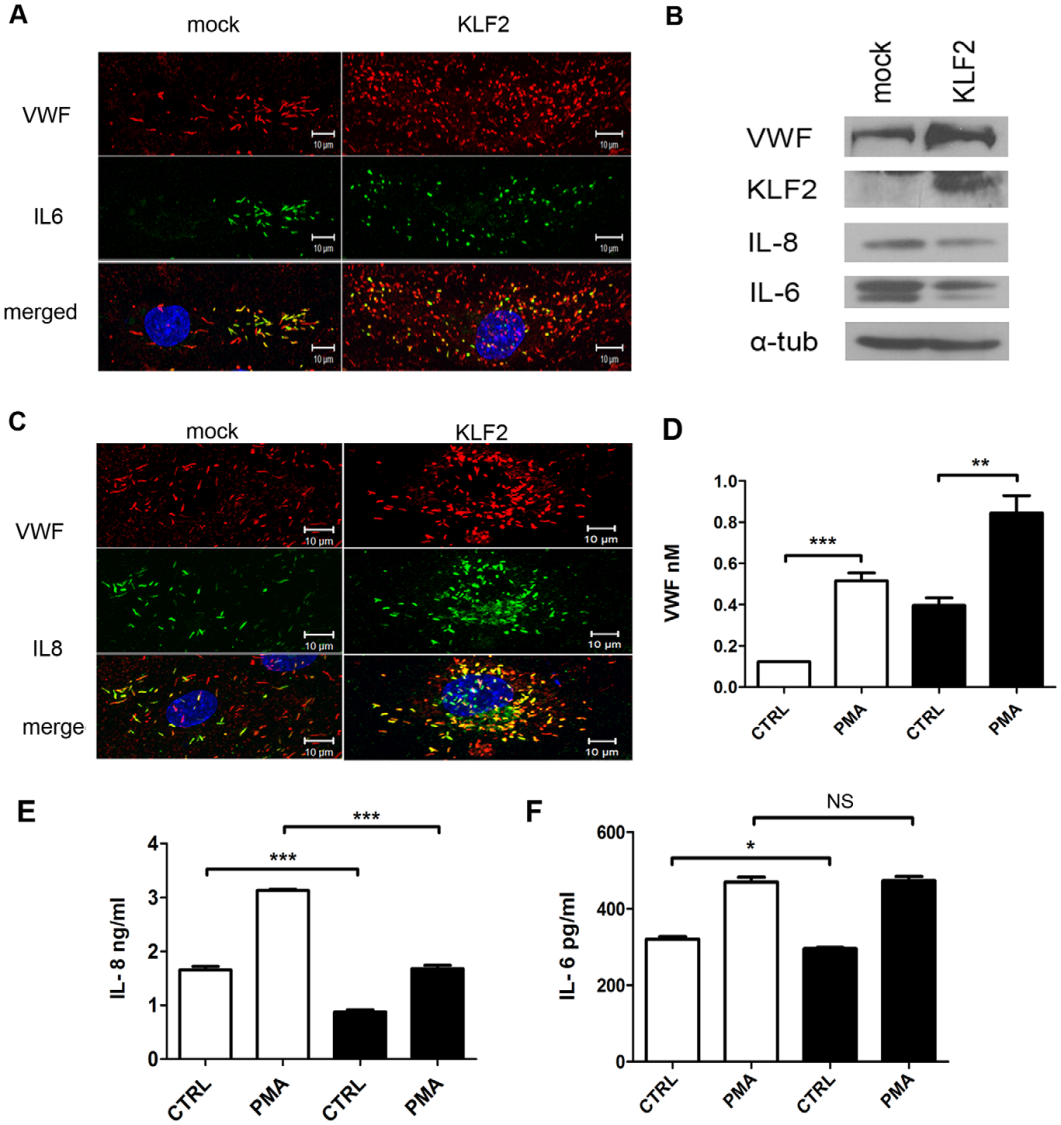
IL-6 and IL-8 content of KLF2-transduced BOECs. (A) Immunofluorescence image showing co-localization of IL-6 (green) and VWF (red) in IL-1β-treated KLF2- and mock-transduced BOECs. Nuclei were visualized with DAPI (blue). Scale bars: 10 µm. (B) Western blot analysis for VWF, KLF2, IL-8 and IL-6 expression in lysates of mock- and KLF2-transduced BOECs; α-tubulin was shown as a loading control. (C) Immunofluorescence image showing co-localization of IL-8 (green) and VWF (red) in IL-1β-treated KLF2- and mock-transduced BOECs. Nuclei were visualized with DAPI (blue). Scale bars: 10 µm. (D) Release of VWF from PMA-stimulated KLF2 (black bars)- and mock (white bars)-transduced cells (IL-1β-treated), measured by determining the concentration of VWF in the conditioned medium by ELISA. **P<0.001; ***P<0.0001 by Students t-test (E-F) Release of IL-6 and IL-8 from PMA-stimulated KLF2 (black bars)- and mock (white bars)-transduced cells (IL-1β-treated), measured by determining the concentration of IL-6 and IL-8 in the conditioned medium by ELISA. The amount of IL-6 released without stimulation was slightly reduced in KLF2 expressing cells when compared to mock-transduced cells. NS: non-significant; *P<0.01; ***P<0.0001 by Students t-test.

The mRNA expression of the proinflammatory protein angiopoietin-2 has previously been shown to be decreased in endothelial cells expressing KLF2 [4]. This prompted us to determine whether Ang2 was present in WPBs of endothelial cells expressing KLF2. First, we addressed the intracellular distribution of Ang2 in mock-transduced endothelial cells. In agreement with previous observations [17] we identified Ang2 in WPBs (Figure 3A). Approximately 20–50% of individual cells displayed Ang2 staining in WPBs. Within a single cell on average 40% of the WPBs stained positive for Ang2. In contrast to previous observations we observed that Ang2 and P-selectin were co-stored in WPBs suggesting that these proteins are not stored in these organelles in a mutually exclusive manner (Figure 3A) [17]. We next addressed Ang2 storage in KLF2-transduced BOEC. In the majority of KLF2-transduced cells, no Ang2 staining was observed (Figure 3C). The decrease in Ang2 expression in KLF2 expressing cells was verified by western blot (Figure 3B). To confirm these findings we performed stimulation experiments using KLF2- and mock-transduced endothelial cells. A two fold increase in secreted Ang2 was observed following stimulation of mock-transduced cells with phorbol 12-myristate 13-acetate (PMA). Conversely, no regulated release of Ang2 was observed in KLF2-transduced endothelial cells (Figure 3E, F). These findings show that expression of KLF2 diminishes the Ang2-content of WPBs. We also monitored the presence of other WPB-components in KLF2-transduced endothelial cells. Osteoprotegerin (OPG) staining was observed in WPBs of both KLF2 and mock-transduced endothelial cells by immunofluorescence staining (Figure 4A). Western blot analysis revealed that OPG protein levels were not affected by KLF2 expression (Figure 4B). A reduced staining was observed for P-selectin but the effect of KLF2 was less pronounced when compared to Ang2 (data not shown). KLF2 has been reported to downregulate IL-1ß-induced upregulation of IL-6 and IL-8 expression. Inspection of IL-1ß treated mock- and KLF2-transduced cells did not reveal major changes in the IL-6 and IL-8 distribution as assessed by confocal microscopy (Figure 5A and C). Western blot analysis of IL-8 and IL-6 however, revealed a slight decrease in expression levels of both proteins upon expression of KLF2 (Figure 5B). To quantitatively address this issue we determined IL-8 and IL-6 release following agonist-induced WPB release. Upon stimulation with PMA a significant reduction in the amount IL-8 was observed in KLF2-transduced cells when compared to mock-transduced cells (Figure 5E). A slightly reduced basal release of IL-6 was observed in KLF2-transduced cells, however, the amount of PMA induced release of IL-6 was similar in KLF2-transduced and mock-transduced cells (Figure 5F). Together our findings show that IL-8 and Ang2 content of WPBs is reduced in endothelial cells expressing KLF2.

### cAMP-mediated Clustering of WPBs is Reduced in KLF2 Expressing BOECs

**Figure 6:**
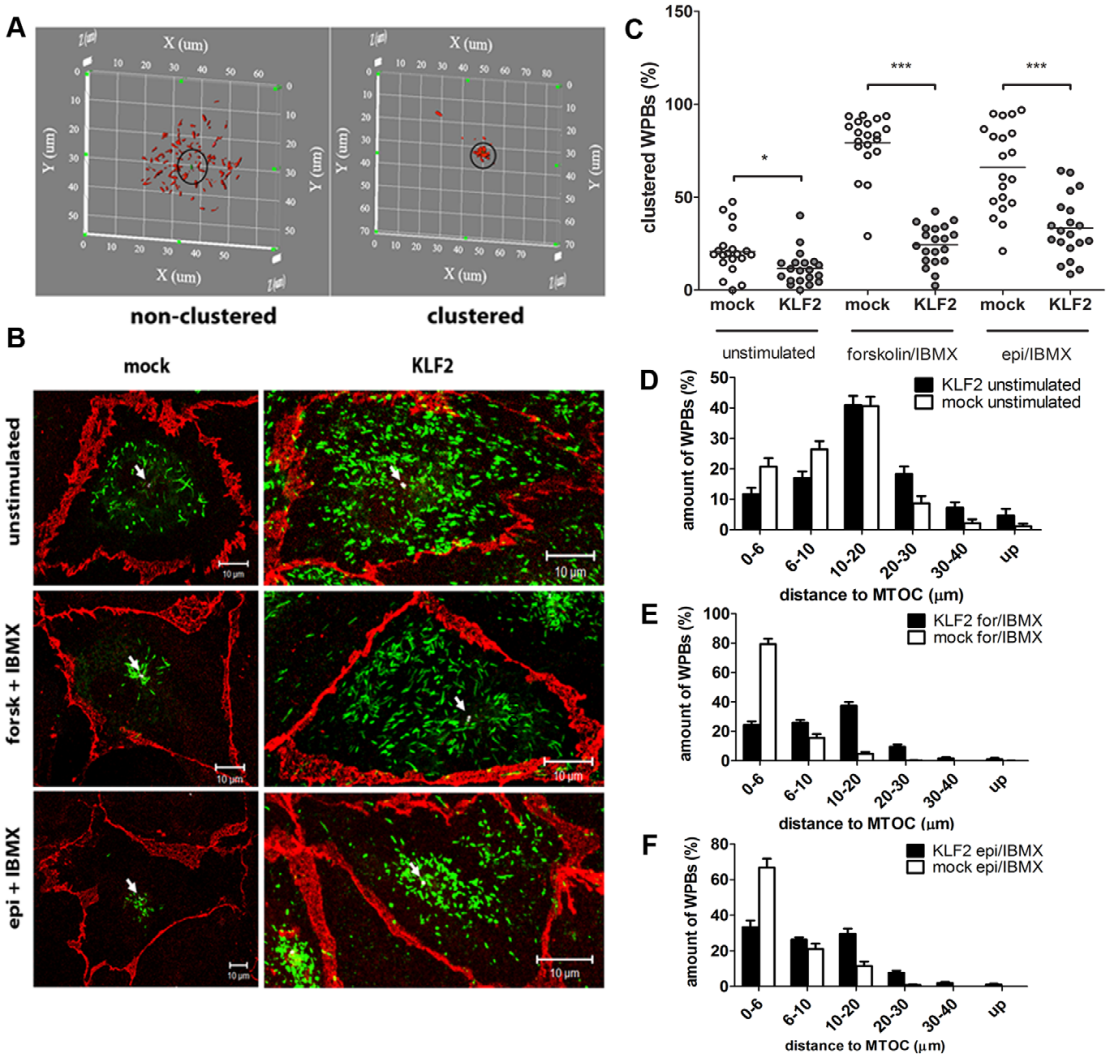
Effects of KLF2 expression on WPB clustering. (A) 3D model of WPB distribution (generated by Image Pro Plus) displaying non-clustered WPBs dispersed through the cell (left panel) and clustered WPB in cells treated with forskolin and IBMX (right panel). A circle with a radius of 6 µm around the MTOC, detected with anti-γ-tubulin antibodies, was used to calculate the percentage of clustered WPBs. (B). Mock- or KLF2-transduced BOECs were stimulated with forskolin + IBMX or epinephrine + IBMX or serum free medium (unstimulated). Following stimulation cells were stained for β-catenin (red), VWF (green) and γ-tubulin (white). The location of the MTOC is indicated by an arrow. Representative confocal images are shown as total intensity projections of the various optical sections acquired per sample (XYZ projections). Scale bars: 10 µm. (C) Mock- or KLF2-transduced BOECs were stimulated with forskolin + IBMX or epinephrine + IBMX or serum free medium (unstimulated) were analyzed for WPB clustering as described in Figure 6A. ***P<0.001 using two-way ANOVA (D-F) Quantification of relative numbers of WPBs and their distance to the MTOC in non-stimulated and stimulated KLF2- and mock-transduced cells. Error bars represent SEM.

Previously, we have shown that WPBs cluster at the MTOC in response to stimulation with cAMP-raising agonists [23], [24]. While performing our initial characterization of KLF2 expressing BOECs we noted a striking absence of clustered WPBs following stimulation with forskolin or epinephrine. This prompted us to investigate a possible role for KLF2 in regulation of clustering by performing a quantitative analysis of WPB clustering in both KLF2- and mock-transduced BOECs. KLF2- or mock-transduced BOECs were stimulated for 1 hour with forskolin and IBMX, epinephrine and IBMX or serum free medium alone as a control. After stimulation, cells were stained for VWF, γ-tubulin and β-catenin. Cells were randomly chosen and analyzed. A ‘clustering’ circular region was defined around the MTOC, wherein WPBs were considered to be clustered. The limit of this region was set at a radius of 6 µm, which is the average length of two longitudinally arranged WPBs (Figure 6A). In mock-transduced BOECs the majority of WPBs were found near the MTOC in response to forskolin and epinephrine stimulation. WPB clustering was respectively 78% and 66% for these two agonists (Figures 6B and C). In unstimulated BOECs the perinuclear WPB clustering was about 21% (Figure 6C). A clear reduction in WPB clustering was observed in BOECs expressing KLF2, where the average percentage of WPB clustering after forskolin and epinephrine stimulation was 24% and 33% respectively (Figures 6B and C). Furthermore, a reduction in the percentage of perinuclear WPB in unstimulated KLF2 cells was observed compared to mock-transduced cells (Figure 6C). The distance of individual WPBs to the MTOC in KLF2- and mock-transduced cells was determined. This analysis revealed that the average distance of WPBs from the MTOC is larger in KLF2-transduced cells when compared to mock-transduced endothelial cells following stimulation with cAMP raising agonists (Figure 6D–F). Together these findings show that perinuclear clustering of WPBs is reduced in KLF2 expressing endothelial cells.

## Discussion

KLF2 is responsible for the shear stress induced modulation of gene expression that limits inflammatory responses and thereby sustains endothelial quiescence [3], [4], [30]. In this study we explored the effects of KLF2 on the dynamics and properties of WPBs. Lentiviral over-expression results in a 20-fold increase of KLF2 as determined by RT-PCR [3]. In comparison, HUVECs grown under laminar flow for 8 hours at a flow rate of 12 dyne/cm^2^, showed a 12-fold increase in KLF2 levels [31]. *In vivo* KLF2 protein levels have been determined in swine endothelium of the atherosusceptible aortic arch (AA) and the atheroresistant descending thoracic aorta (DT); a 1.5-fold increase in KLF2 protein levels were found in the DT as compared to the AA [32]. However, it was reported that pathological hepatic hemodynamic variations occurring during liver cirrhosis development caused a 6-fold increase of KLF2 protein levels *in vivo* [33].

In agreement with previous observations we show that the number of secretable WPBs increases upon expression of KLF2 [3]. We anticipate that the increased number of WPBs results from the upregulation of VWF mRNA levels induced by KLF2 [3]. The increase in number of WPBs in KLF2-transduced cells suggests that generation of WPBs is mainly driven by the amount of VWF synthesized by endothelial cells. It has been well-established that the formation of WPBs is strictly dependent on the presence of VWF [34]. Absence of WPBs affects the regulated release of other WPB constituents such as P-selectin. An elegant study revealed that the absence of releasable P-selectin in VWF deficient mice impairs leukocyte recruitment [35]. Based on these findings we anticipate that increased numbers of WPBs in KLF2-transduced cells can potentially enhance the storage capacity of other WPB components. We observed that the average WPB length in KLF2-transduced cells is consistently reduced by 0.4 µm when compared to mock-tranduced cells (Figure 2). Biogenesis and elongation of WPBs is mediated by a clathrin/AP-1 coat that transiently associates with newly forming WPB in the TGN [36]. We speculate that expression of KLF2 modulates the function or expression of these proteins thereby limiting the elongation of newly forming WPBs. The overall multimeric composition of secreted VWF in medium of KLF2- and mock-transfected BOECs was similar, suggesting that the observed decrease in WPB length is not due to a change in multimerization of VWF. (Figure S1). However, our analysis did not allow us to assess whether the maximum size of VWF multimers is affected by the expression of KLF2.

Upon stimulation of endothelial cells with cAMP-raising agonists a significant number of WPBs escape from their release and are transported to the MTOC [23]. Here, we show that clustering of WPBs at the MTOC is impaired in KLF2 expressing endothelial cells (Figure 6). Clustering of WPBs is dependent on dynein-mediated transport along microtubules [24]. When laminar fluid shear stress is applied microtubules align in the direction of the flow and the MTOC migrates downstream of the nucleus [37]. Expression of KLF2 does not result in major changes in the properties of microtubules nevertheless clustering of WPBs is impaired in these cells. Despite the absence of clustering at the MTOC a considerable number of WPBs is retained inside the KLF2 expressing cells that have been triggered by cAMP-raising agonists (Figure 2). We speculate that defects in minus end directed transport of WPBs along microtubules prevent perinuclear transport under these conditions. It has been found previously that the retrograde transport of WPBs is mediated by the dynein-dynactin complex and that protein kinase A (PKA) is involved in this process, as inhibition of PKA prevents clustering of WPBs at the MTOC. It is unlikely that KLF2 targets activation of PKA as it has been described previously that the sensitivity of KLF2 expressing endothelial cells for cAMP-raising agonists like epinephrine and forskolin is not reduced [3]. We speculate that KLF2 modulates PKA-mediated phosphorylation of motor protein complexes regulating retrograde transport of WPBs. We previously proposed that clustering of WPBs at the MTOC might be a mechanism to secure vascular homeostasis by preventing release of pro-inflammatory contents from WPBs [24].

Previously, it was reported that the storage of Ang2 and P-selectin in WPBs was mutually exclusive [17]. In this study, we have shown that Ang2 and P-selectin co-localize in WPBs. P-selectin is able to bind to the D’D3 domain of VWF [38] via its luminal domain, however it was also reported that a targeting motif in the cytoplasmic domain of P-selectin was sufficient for sorting of P-selectin to WPBs [39]. This makes mutual exclusion by competitive binding of P-selectin and Ang2 to VWF unlikely.

KLF2 is known to cause downregulation of expression of pro-inflammatory cytokines, some of which can be present in the WPBs [3], [40]. This is supported by the finding that the amount of Ang2 and (to a lesser extent) IL-8 in the WPBs is reduced in KLF2 expressing endothelial cells (Figure 3 and 5). While angiopoietin-1 (Ang1)/Tie-2 signaling promotes vascular quiescence, the pro-inflammatory protein Ang2 destabilizes the vasculature at sites of vessel remodeling by antagonizing the binding of Ang1 to Tie-2 [41]. Ang2 primes the endothelium to respond to pro-inflammatory cytokines, such as VEGF, TNF-α and IL-1β [42]. In this manner Ang2 triggers an inflammatory response by activating the endothelium, inducing endothelial permeability and extravasation of inflammatory cells [43] and thereby contributes to premature atherosclerosis. A three- to four-fold decline in Ang2 levels induced by flow (30 dyne/cm^2^) has been reported [44]. The observed decline in Ang2 levels in KLF2-transduced endothelial cells is consistent with this observation. Our findings show that KLF2-transduced cells do not contain a rapidly releasable reservoir of Ang2. Recently, VWF has also been implicated in the regulation of angiogenesis [45]. Absence of VWF resulted in a two-fold release in Ang2 levels in culture supernatants [45]. As Ang2 is co-stored with VWF in WPBs; in the absence of VWF Ang2 is released in a constitutive manner. We anticipate that targeting of Ang2 to WPBs provides a mechanism to reduce circulating levels of Ang2 thereby promoting vascular quiescence. In the absence of WPBs, due to lack of VWF, Ang2 levels increase and destabilize the vasculature allowing for vascular remodeling [41].

Reduced expression of Ang2 by endothelial cells, decreases inflammation and vascular remodeling by making the endothelium less responsive to pro-inflammatory cytokines. The decrease in secreted IL-8 from IL-1β stimulated KLF2 cells might result from this decreased sensitivity to cytokines; however the amount of secreted IL-6 is still the same in KLF2 cells. These findings suggest that KLF2 expressing endothelial cells are less prone to vascular remodeling, inflammation and ultimately atherosclerosis. In contrast, at sites of disturbed flow, where endothelial cells do not express KLF2, Ang2 can be released from the WPBs, promoting inflammation and vascular remodeling and therefore making these sites more susceptible to premature atherosclerosis and plaque neovascularization. Our findings on the lack of Ang2 in KLF2-transduced endothelial cells together with previous observation on down-regulation of Ang2 levels by flow provide a mechanism to stabilize newly formed vasculature by reducing the synthesis of vessel-destabilizing Ang2.

## Supporting Information

Figure S1 **VWF multimer gel. Secreted VWF in medium of PMA-induced mock- and KLF2-transduced BOECs.** The multimerization patterns of mock and KLF2 samples appear to be similar.
(TIF)
