# Supplementary figures and images for: The Shear Stress-Induced Transcription Factor KLF2 Affects Dynamics and Angiopoietin-2 Content of Weibel-Palade Bodies
Source: PLoS One. 2012 Jun 8;7(6):e38399. doi: 10.1371/journal.pone.0038399 (PMC3371018; doi:10.1371/journal.pone.0038399)

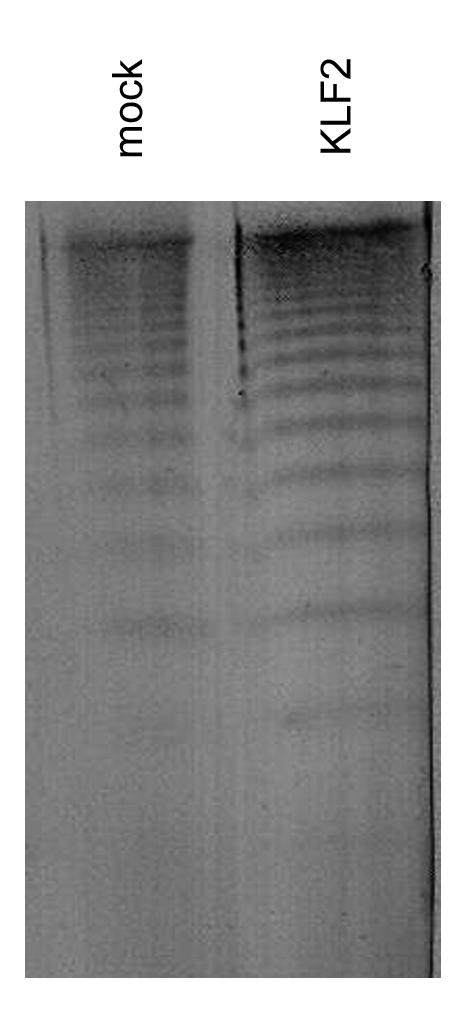

Supplement: Figure S1 — VWF multimer gel. Secreted VWF in medium of PMA-induced mock- and KLF2-transduced BOECs. The multimerization patterns of mock and KLF2 samples appear to be similar. (TIF) [file pone.0038399.s001.tif]
